# DNA metabarcoding adds valuable information for management of biodiversity in roadside stormwater ponds

**DOI:** 10.1002/ece3.5503

**Published:** 2019-08-02

**Authors:** Zhenhua Sun, Markus Majaneva, Ekaterina Sokolova, Sebastien Rauch, Sondre Meland, Torbjørn Ekrem

**Affiliations:** ^1^ Architecture and Civil Engineering, Water Environment Technology, Chalmers University of Technology Gothenburg Sweden; ^2^ Department of Natural History Norwegian University of Science and Technology, NTNU University Museum Trondheim Norway; ^3^ Faculty of Environmental Sciences and Natural Resource Management Norwegian University of Life Sciences Ås Norway; ^4^ Norwegian Institute for Water Research (NIVA) Oslo Norway

**Keywords:** DNA metabarcoding, high throughput sequencing, macroinvertebrates, pollutants, road runoff, stormwater ponds

## Abstract

**Abstract:**

1. Stormwater ponds are used to compensate for the adverse effects that road runoff might have on the natural environment. Depending on their design and placement, stormwater ponds can act as both refugia and traps for local biodiversity. To evaluate the impact of stormwater ponds on biodiversity, it is critical to use effective and precise methods for identification of life associated with the water body. DNA metabarcoding has recently become a promising tool for identification and assessment of freshwater biodiversity.
2. Using both morphology and DNA metabarcoding, we analyze species richness and biological composition of samples from 12 stormwater ponds and investigate the impact of pond size and pollution levels in the sediments and water column on the macroinvertebrate community structure.
3. DNA metabarcoding captured and identified more than twice the number of taxa compared to morphological identification. The (dis)similarity of macroinvertebrate community composition in different ponds showed that the ponds appear better separated in the results obtained by DNA metabarcoding, but that the explained variation is higher for the results obtained by morphologically identification, since it provides abundance data.
4. The reliance on morphological methods has limited our perception of the aquatic biodiversity in response to anthropogenic stressors, thereby providing inaccurate information for appropriate design and management of stormwater ponds; these drawbacks can be overcome by DNA metabarcoding.
5. *Synthesis and applications*. The results indicate that DNA metabarcoding is a useful tool in identifying species, especially Diptera, which are difficult to determine. Application of DNA metabarcoding greatly increases the number of species identified at each sampling site, thereby providing a more accurate information regarding the way the ponds function and how they are affected by management.

**OPEN PRACTICES:**

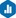

This article has earned an Open Data Badge for making publicly available the digitally‐shareable data necessary to reproduce the reported results. The data is available at https://www.ebi.ac.uk/ena/data/view/PRJEB30841.

## 1 INTRODUCTION

Roads may cause harmful effects on aquatic ecosystems, due to alternations in hydrological processes (Karlson & Mörtberg, 2015) and pollutants from vehicles, asphalt, and operation and maintenance activities (Meland et al., 2011; Meland, Salbu, & Rosseland, 2010). The awareness that roads may significantly impair the aquatic environment has resulted in the construction of an increasing number of stormwater ponds along roads. Several studies have reported on the biodiversity services the stormwater ponds provide as anthropogenic habitats, enhancing urban biodiversity (e.g., Hassall, 2014; Hill et al., 2017; Le Viol, Chiron, Julliard, & Kerbiriou, 2012; Stephansen, Nielsen, Hvitved‐Jacobsen, Pedersen, & Vollertsen, 2016). However, some studies, for example, Helfield and Diamond (1997), question such uses of stormwater ponds, mainly since their primary function of improving water quality leads to the presence and bioavailability of pollutants. In order to clarify the biodiversity value of stormwater ponds and improve the management of stormwater systems to support biodiversity, effective methods should be applied to determine effects of pollutants on the biota.

Current research on aquatic biodiversity in stormwater ponds is typically based on identification of organisms by morphological properties (Briers, 2014; Le Viol, Mocq, Julliard, & Kerbiriou, 2009; Tixier, Rochfort, Grapentine, Marsalek, & Lafont, 2012). However, identification/sorting of invertebrate using morphological properties to species level is difficult and expensive (Baloğlu, Clews, & Meier, 2018; Wang, Srivathsan, Foo, Yamane, & Meier, 2018). It has been shown that the cost of, for example, midge, identification using morphological properties is quite high due to the requirement of dissection and mounting of specimens onto microscopic slides (Baloğlu et al., 2018). As a result, the rarely used species‐level data lead to the undesirable information of responses to stressors, because different species have different sensitivity to environmental parameters (Baloğlu et al., 2018; Beermann, Zizka, Elbrecht, Baranov, & Leese, 2018; Macher et al., 2015).

Recently, DNA‐based identification methods have gained popularity in overcoming the limitations of morphological identification. DNA metabarcoding allows rapid and cost‐effective assessment and monitoring of biodiversity using massive parallel sequencing of bulk samples (Cristescu, 2014; Leese et al., 2018). Several studies have applied and compared DNA metabarcoding with morphological identification in assessments of macroinvertebrate diversity (Chan et al., 2014; Elbrecht & Leese, 2017; Renaud, Savage, & Adamowicz, 2012). Beermann et al. (2018) indicated that although, for example, in the case of chironomids, most operational taxonomic units (OTUs) (>85%) do not match with high similarity (>98%) to formally described species due to incomplete reference libraries, DNA‐based approaches are still potentially useful when studying environmental impacts, especially in the context of multiple stressors.

A major challenge in (PCR‐based) metabarcoding is that it does not provide quantitative abundance data, especially in the case of diverse community samples (Elbrecht & Leese, 2015). Several studies have detected significant linear relationships between the number of morphologically identified specimens and the number of sequencing reads but with poor fit and high scatter (Carew, Pettigrove, Metzeling, & Hoffmann, 2013; Clarke, Beard, Swadling, & Deagle, 2017; Elbrecht, Vamos, Meissner, Aroviita, & Leese, 2017). This means that DNA metabarcoding data can be used only as a semi‐quantitative abundance estimator. In addition, if the specimen in the reference library has been wrongly identified or not updated according to the latest taxonomic revision, a match will be assigned to the wrong name (Pawlowski et al., 2018).

This study is focused on testing the following hypothesis: DNA metabarcoding can provide better results and explanation than morphological identification in identifying organisms to species level and studying environmental impacts. Macroinvertebrates in stormwater ponds were identified by both morphology and DNA metabarcoding, allowing us to (a) compare the (dis)similarity of macroinvertebrate community composition in different ponds using the data obtained by morphological identification and DNA metabarcoding; (b) compare the differences in species richness of groups in common using the data obtained by morphological identification and DNA metabarcoding; and (c) compare the power of morphological identification and DNA metabarcoding in predicting the impacts of environmental variables on the macroinvertebrate community composition.

## 2 MATERIALS AND METHODS

Twelve stormwater ponds were investigated. The ponds are located along the major highways E6 and E18 in the counties of Oslo, Akershus, and Østfold in southern Norway (Figure S1).

### 2.1 Biological sampling

Biological samples were collected four times (April, June, August, and October) in 2013. The samples were collected with a kick net and traps close to the inlet and twice on either side of the main basin within each pond. A kick net with a 30 × 30 cm opening and a mesh size of 0.45 mm was used. Kick sampling with five sweeps was applied, when there were small stones at the bottom; five sweeps were taken through the water and aquatic vegetation at approximately 50 cm depth, when the bottom material was not stony. Traps made of 1.5 L transparent plastic bottles were also used to collect samples (Sun et al., 2018); the traps were left for 1–4 days, according to time of the year. The samples were preserved in 70% ethanol.

Morphological identification was performed by sorting organisms in the laboratory and identifying them to species level when possible using morphological characteristics. Nilsson (1996, 1997) was used to identify benthic macroinvertebrates; Nilssen (1974) was used to identify Chaoboridae.

The morphologically identified specimens were collected in one glass vial per sample. The samples were preserved in 70% ethanol and stored at +5°C. DNA was extracted by first pouring the specimens onto Petri dishes, carefully pipetting away excess ethanol, and drying the samples overnight until the specimens were completely dry. The dried specimens were collected in sterile mortar and ground with a pestle in liquid nitrogen. The obtained tissue powder was transferred to microcentrifuge tubes and stored at −20°C. From each sample, 10–20 mg subsamples were taken in triplicate to DNeasy 96 plates where DNA was extracted using DNeasy 96 Blood & Tissue Kit (Qiagen GmbH). The samples were incubated with proteinase K and buffer ATL at 56°C overnight, and the subsequent steps were done according to the manufacturer's protocol, except DNA was eluted in 2 × 200 µl of DNase‐free water.

Two fragments of the mitochondrial COI gene were amplified three times from each three DNA extraction subsamples. The approximately 230 bp long fragment at the 5’ end of the barcoding region was amplified, using the LCO1490 (Folmer, Black, Hoeh, Lutz, & Vrijenhoek, 1994) forward primer (GGTCAACAAATCATAAAGATATTGG) and the 230_R (Gibson et al., 2015) reverse primer (CTTATRTTRTTTATICGIGGRAAIGC). The approximately 420 bp long fragment at the 3’ end of the barcoding region was amplified, using the forward primer BF2 (GCHCCHGAYATRGCHTTYCC) and the reverse primer BR2 (TCDGGRTGNCCRAARAAYCA) described by Elbrecht and Leese (2017). The amplifications were carried out with Illumina forward (TCGTCGGCAGCGTCAGATGTGTATAAGAGACAG) and reverse (GTCTCGTGGGCTCGGAGATGTGTATAAGAGACAG) adapters.

The PCR and bioinformatic processing were conducted as described in Majaneva, Diserud, Eagle, Hajibabaei, and Ekrem (2018). One exception is that for the reads amplified with BF2 and BR2 primers, all reads shorter than 430 and longer than 490 bases were removed. The exact commands are provided in the Appendix S1.

### 2.2 Environmental variables

Water and sediment samples were collected once in April 2013. Water samples were collected close to the inlet of the ponds. Sampling was performed using acid washed 125 ml polyethylene (PE) bottles, including acid washed bottles for metal analysis. Top‐layer sediments were collected close to the inlet using a spade and stored in 1 L glass bottles. Since the highest contaminant concentrations are typically found in the inlet (Allen, Haynes, & Arthur, 2017), the samples serve as a proxy for detecting contamination within the ponds.

All sediment and water quality analyses were performed by Rambøll Analytics Laboratories in Finland. Fourteen water quality variables were analyzed in this study, including metals (aluminum (Al), barium (Ba), potassium (K), calcium (Ca), copper (Cu), magnesium (Mg), manganese (Mn), silicon (Si), iron (Fe), and titanium (Ti)), chloride (Cl^‐^), total nitrogen (TN), total phosphorus (TP), and sulfate (SO_4_^2‐^). Eleven sediment variables were analyzed, including total organic carbon (TOC), total hydrocarbons, 16 polycyclic aromatic hydrocarbons (US EPA 16 PAHs), aluminum (Al), calcium (Ca), chromium (Cr), copper (Cu), iron (Fe), nickel (Ni), lead (Pb), and zinc (Zn). In the subsequent statistical analysis, pyrene was used as a proxy for PAH pollution, because the concentrations of most other PAH compounds were below the limit of quantification (LOQ) in more than 15% of the total number of samples.

Pond size, which ranges from 89 to 1,400 m^2^, was obtained from the Norwegian Public Roads Administration (NPRA) (Table S1).

### 2.3 Statistical analyses

The number of species from groups in common between DNA metabarcoding and morphological identification was compared using bar charts generated with R software (R Core Team, 2013).

Principal coordinates analysis (PCoA) and distance‐based redundancy analysis (db‐RDA) were performed on the datasets obtained using both DNA metabarcoding and morphological identification. PCoA was used to compare the (dis)similarity of whole macroinvertebrate community composition in different ponds. db‐RDA was used to explore the impacts of environmental variables on the macroinvertebrate community composition of groups in common of the two methods, aiming to compare the power of morphological identification with that of DNA metabarcoding. Groups that were included in the db‐RDA were Gastropoda, Odonata, Ephemeroptera, Plecoptera, Trichoptera, Lepidoptera, Megaloptera, Hemiptera, Diptera, Coleoptera, Oligochaeta, and Hirudinea. Using morphology, some macroinvertebrates were only identified to genus or (sub)family level. DNA metabarcoding can only provide reliable data for presence/absence, while morphological identification can provide abundance data. Therefore, both abundance and presence/absence of each taxon were used in the morphological dataset, while presence/absence of each species was used in the DNA metabarcoding dataset, in which 1 represents presence and 0 represents absence. The abundance data were log (*x* + 1) transformed before PCoA and db‐RDA to increase the influence of subordinate species; otherwise, the results would be completely dependent on the dominant species (Šmilauer & Lepš, 2014). PCoA was used to calculate a distance matrix and represent the (dis)similarity measures in the Euclidean space; the (dis)similarity can then be assessed using linear models (Legendre & Anderson, 1999). The distances between points in the plot are close to original dissimilarities. Two similarity measures were used: Sørensen binary distance (Sørensen, 1948) for presence–absence data and Bray–Curtis distance (Bray & Curtis, 1957) for quantitative data (abundance); the equations are defined in the Appendix S1. In order to provide a better overview of correlation between different methods, Pearson's correlation analysis was applied using PCoA scores extracted from the first four ordination axes, and a matrix of the correlation coefficients was generated using R software (R Core Team, 2013).

Principal component analysis (PCA) was performed on both the sediment and water quality data to reduce the skewness and improve the normality of the data for the db‐RDA, and the data were log(*x *+ 1) transformed prior to the PCA. PCA sample scores extracted from axes 1 and 2 were used to represent pollution levels in the water column and sediments, respectively (Figure S2). PCA, PCoA, and db‐RDA were performed using CANOCO 5 software (Canoco5, 2012). Monte Carlo permutation tests (999 permutations, *p* < .05) were used to determine the statistical significance. The species that showed correlation with ordination axes smaller than −0.6 or larger than 0.6 were displayed in the plots in order to avoid the ordination plots becoming too cluttered.

## 3 RESULTS

### 3.1 Comparison of species richness

Using morphological identification, a total of 156 macroinvertebrates were identified, of which 88 (56%) were identified to the species level and 68 (44%) were identified either to the genus or (sub)family level. Four macroinvertebrates are red‐listed species in Norway, that is, *Planorbis planorbis* (deficient category), *Coenagrion lunulatum* (vulnerable category), *Orthetrum cancellatum* (vulnerable category), and *Chaoborus pallidus* (threatened category) (Henriksen & Hilmo, 2015). Among these four species, only one was identified using DNA metabarcoding.

Using DNA metabarcoding, collectively for all samples, a total of 10.7 and 12.9 million reads were generated from the F230 and BFR2 fragments, respectively. After quality control and merging the taxonomic information from the two fragments, 599 OTUs were identified to invertebrate taxa, most of which were species, but some were genus or family level, because the read matched several species with a high similarity value. In the final dataset, the species that had five or less reads in one sample were excluded. As a result, 342 OTUs from benthic macroinvertebrates were included in the statistical analysis. DNA metabarcoding captured and identified more than twice the number of taxa compared to identification by morphology across the 12 study ponds. A total of 196 (57%) OTUs could be unambiguously associated with a species name in the barcode reference library, while 146 (43%) were left without species‐level identification due to the low percentage match for local species or due to matching multiple species with same percentage.

**Figure 1:**
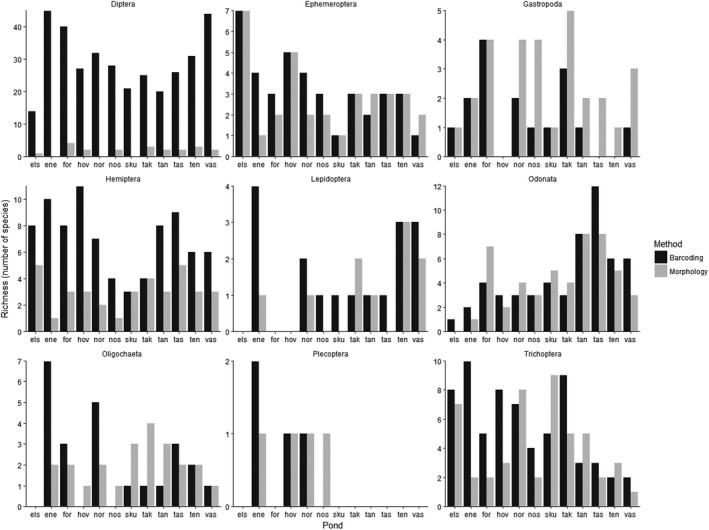
Comparison of the number of species detected by DNA metabarcoding and morphology in the taxonomic groups in common. The abbreviations along the x‐axis represent names of ponds: els‐Elstadmoen, ene‐Enebekk, for‐Fornebu, hov‐Hovinmoen, nor‐Nordby, nøs‐Nøstvedt, sku‐Skullerud, tak‐Taraldrud crossing, tan‐Taraldrud north, tas‐Taraldrud south, ten‐Tenor, and vas‐Vassum

Both identification methods detected Oligochaeta, Diptera, Ephemeroptera, Gastropoda, Lepidoptera, Hemiptera, Odonata, Plecoptera, and Trichoptera; the number of species from these groups was compared. Identification of species by DNA metabarcoding resulted in considerably more Diptera species than identification by morphology in all ponds (Figure 1), and 66% of all Diptera belonged to the large and ecologically diverse family Chironomidae. There is no comprehensive literature that can be used to identify aquatic Diptera to species level using morphology. This is especially true for the species‐rich family Chironomidae, in which many species remain undescribed in the larval stage in our study, and early instar larvae are usually impossible to identify to species morphologically.

However, COI gene‐based metabarcoding may not be applicable to identify all macroinvertebrate species. In our study, the identification by morphology detected more species of Gastropoda than DNA metabarcoding, and the number of species in some groups, for example, Oligochaeta and Odonata, in a few ponds were higher using morphology (Figure 1).

### 3.2 (Dis)similarity of macroinvertebrate community composition in stormwater ponds

**Figure 2:**
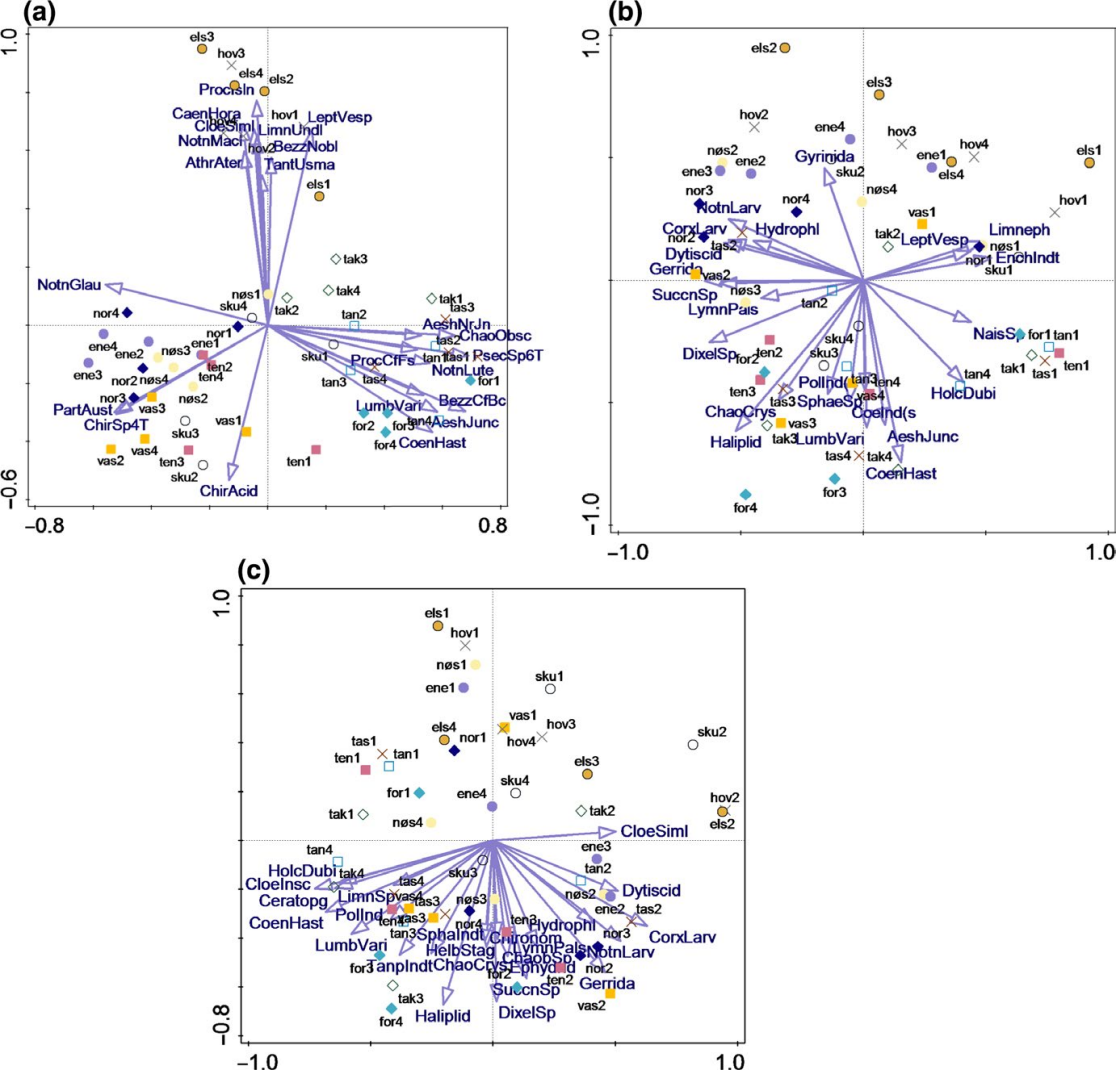
(a) Principal coordinates analysis for the DNA metabarcoding dataset. (b) Principal coordinates analysis for morphological methods based on presence/absence. (c) Principal coordinates analysis for morphological methods based on abundance. The abbreviations on points represent names of ponds (see caption Figure 1); “1,” “2,” “3,” and “4” indicate that the samples were collected in April, June, August, and October, respectively

PCoA was applied to the full datasets obtained from morphological identification and DNA metabarcoding to compare the (dis)similarity of macroinvertebrate community composition in different ponds. The PCoA result for the DNA metabarcoding dataset showed that the species composition in the ponds Hovinmoen and Elstadmoen were quite different from other study ponds (Figure 2a). According to the distance matrix, the first two axes explained 7.4% and 6.6% of the variation, respectively. The organisms were divided into several clusters. Certain species, for example, *Caenis horaria*, *Leptophlebia vespertina,* and *Athripsodes aterrimus*, formed one cluster, and they can be found either in pond Elstadmoen or Hovinmoen. Another group formed by *Aeshna juncea*, *Lumbriculus variegatus,* and allies were mainly found in pond Taraldrud crossing, Taraldrud south, or Fornebu. For the morphological data, that included abundance, the first two axes explained 13% and 12% of the variation in the distance matrix, respectively. For the morphological data based on presence/absence only, the first two axes explained 6.6% and 5.2%, respectively (Figure 2b). The PCoA results for morphological dataset based on abundance showed that the community composition in some ponds at certain time, for example, Hovinmoen (April, August, and October), Enebekk (April and October), Nordby (April) and Nøstvedt (April and October), were similar (Figure 2c). The taxa that are displayed below the x‐axis on the graph could not be found or had low abundance in the ponds above the x‐axis.

**Figure 3:**
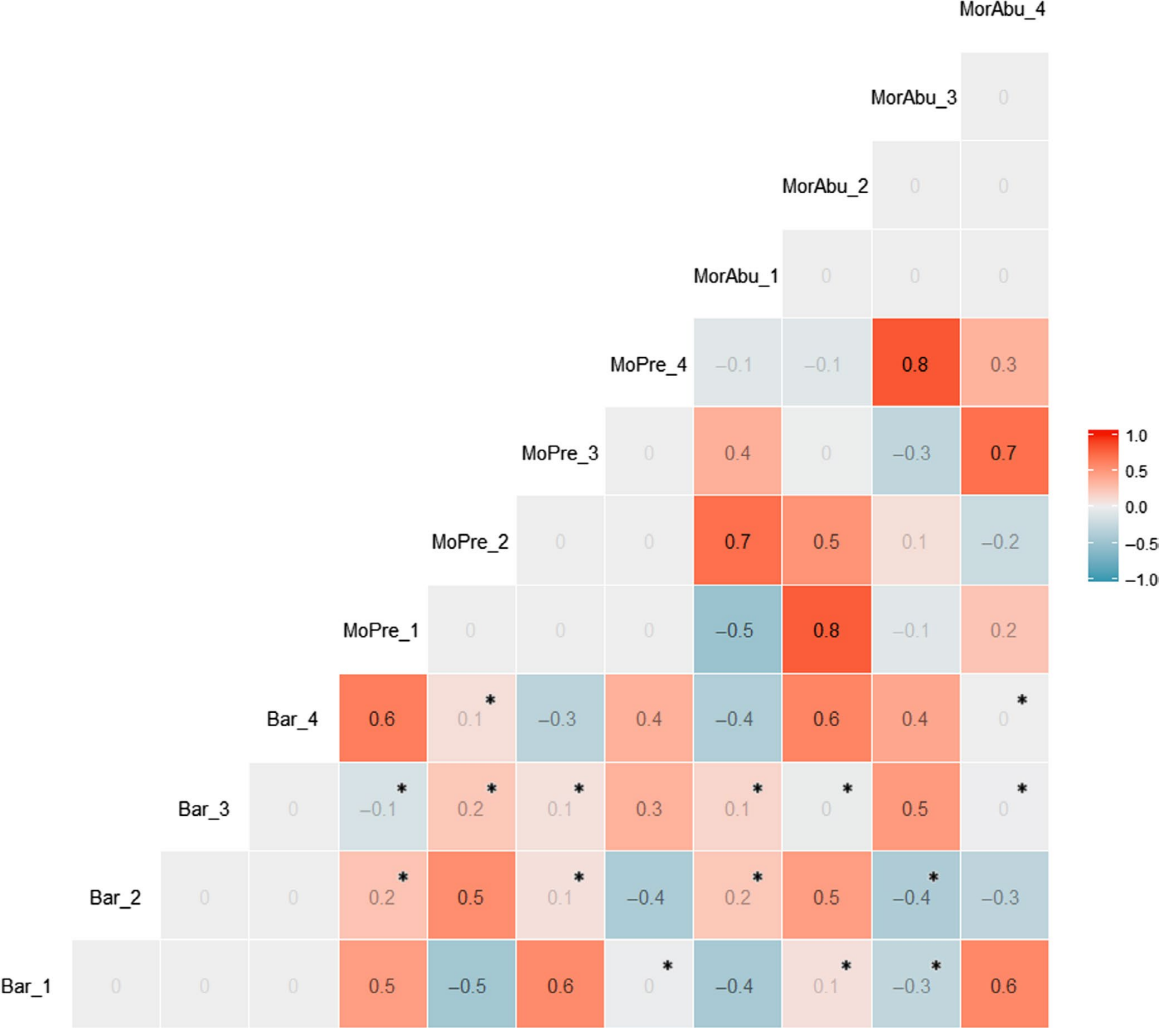
The Pearson correlation matrix of principal coordinates analysis (PCoA) scores extracted from the first four axes in three datasets, including DNA metabarcoding, morphological identification based on presence/absence, and morphological identification based on abundance. “Bar_1,” “Bar_2,” “Bar_3,” and “Bar_4” represent PCoA scores extracted from axes 1, 2, 3, and 4 from DNA metabarcoding dataset, respectively; “MoPre_1,” “MoPre_2,” “MoPre_3,” and “MoPre_4” represent PCoA scores extracted from axes 1, 2, 3 and 4 from morphological identification based on presence/absence dataset, respectively; “MorAbu_1,” “MorAbu_2,” “MorAbu_3,” and “MorAbu_4” represent PCoA scores extracted from axes 1, 2, 3, and 4 from morphological identification based on abundance, respectively. “*” represents the nonsignificant correlations between DNA metabarcoding and morphological identifications

The matrix of the correlation coefficients for the PCoA scores (Figure 3) revealed that PCoA scores extracted from the first axis of DNA metabarcoding were strongly correlated with PCoA scores extracted from the first three axes of morphological identification based on presence/absence (*r* = .5, −.5 and .6, respectively, all *p* < .001). However, most of the PCoA scores extracted from the first four axes of DNA metabarcoding and morphological identification based on abundance were not significantly correlated.

### 3.3 Comparison of the power of DNA metabarcoding and morphological identification in predicting the impacts of environmental variables

**Figure 4:**
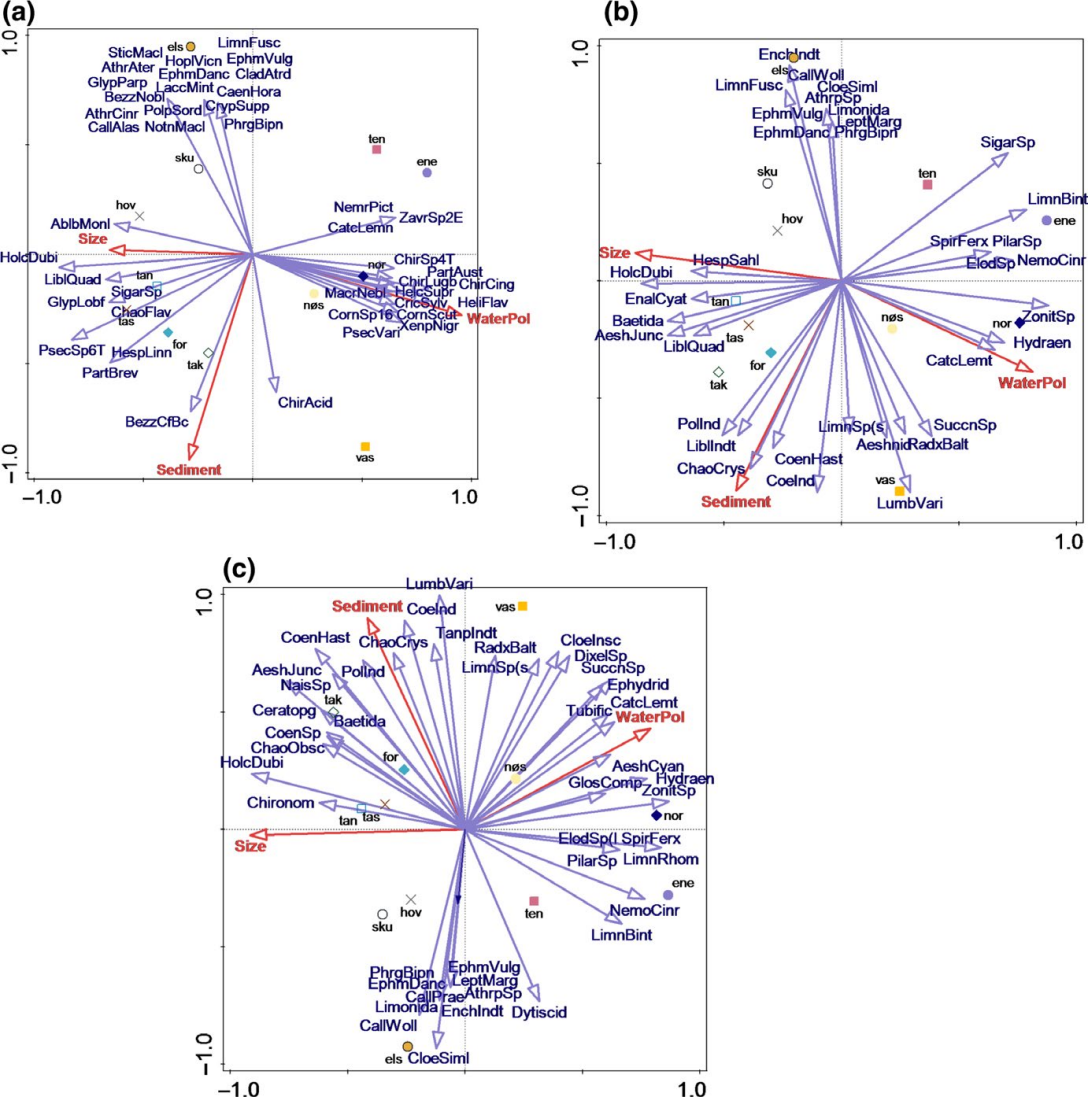
The relationship between macroinvertebrate community composition and pollution levels in the water column and sediments, as well as pond size. (a) Results obtained from db‐RDA for the DNA metabarcoding dataset. (b) Results obtained from db‐RDA for the morphological dataset based on presence/absence. (c) Results obtained from db‐RDA for the morphological dataset based on abundance. The purple arrows represent different species; the red arrows represent environmental variables, in which WaterPol and Sediment represent pollution levels in the water column and sediments, respectively

The results of db‐RDA for the DNA metabarcoding dataset showed that pollution levels in the water column and sediments, as well as pond size, explained 32.2% of variances (first axis: *F* = 1.3, *p* = .02; all axes: *F* = 1.3, *p* = .01) in the macroinvertebrate community composition, and the first two axes explained 14% and 9%, respectively (Figure 4a). For the morphological dataset based on presence/absence, the three variables explained 34.4% of variances (first axis: *F* = 1.3, *p* = .081; all axes: *F* = 1.4, *p* = .004), and the first two axes explained 14% and 13%, respectively; for the morphological dataset based on abundance, these three environmental variables explained 36.6% of variances (first axis: *F* = 1.8, *p* = .07; all axes: *F* = 1.5, *p* = .023), and the first two axes explained 18% and 14%, respectively. However, the first axis based on datasets derived from morphological identifications was not significantly correlated with the macroinvertebrate community composition (Figure 4b,c). Similar to the results of PCoA, the abundance dataset explained most of the variation, followed by presence/absence dataset based on morphological identification. The DNA metabarcoding dataset explained the least variation, a consequence of increased complexity when the number of species increases, because species responds differently. In addition, it is normal that the explained variation in first axes is reduced when the number of response variables is increased. As an example, when DNA metabarcoding was applied to a species‐rich group, for example, Diptera, it provided better results, which explained the highest variation in the macroinvertebrate community composition (Figure S3).

DNA metabarcoding‐based results showed that the taxa were divided into several obvious clusters (Figure 4a), while the morphology‐based results showed that the taxa were distributed all over the space (Figure 4b,c). For the DNA metabarcoding dataset, 26 dipteran species were exhibited in the plot; 12 of these were positively correlated with the pollution level in the sediments, while 14 were negatively correlated. Similarly, 13 dipteran species were positively correlated with pollution level in the water column, while 13 were negatively correlated. Different from DNA metabarcoding‐based results, morphology‐based results based on abundance showed that most of the displayed dipteran taxa (seven out of nine) were positively correlated with pollution level in the sediment. However, five of the dipteran taxa that were positively correlated with pollution level in the sediment were only identified to the higher taxonomic levels: one was identified to genus level (Dixella), one was identified to subfamily level (Tanypodinae), and three were identified to family level (Ceratopogonidae, Chironomidae, and Ephydridae). Only two were identified to the species level, *Chaoborus crystallinus* and *Chaoborus obscuripes*, and they were recognized by both DNA metabarcoding and morphology, and had the same responses to environmental variables according to both methods (Figure 4), that is, they were positively correlated with pond size and pollution level in the sediments, but negatively correlated with pollution level in the water column.

Morphological identification including abundance showed that among the taxa that were negatively correlated with the pollution level in sediments, 43% belonged to Ephemeroptera, Plecoptera, and Trichoptera, and most of them were identified to the species level. Several species of Trichoptera and Ephemeroptera were also found by DNA metabarcoding, and they exhibited the same responses to the environmental variables as morphological identification. For instance, *Phryganea bipunctata, Ephemera vulgata, Ephemera danica,* and *Limnephilus fuscinervis* were all positively correlated with pond size and negatively correlated with the pollution levels in the sediments and water column.

Odonata (dragonflies (Anisoptera) and damselflies (Zygoptera)) and Hemiptera were the groups shown in the plots for both datasets. Common species, such as *Libellula quadrimaculata*, exhibited the same responses to the environmental variables for the two methods, in which they were positively correlated with the pond size and pollution level in the sediment, but negatively correlated with the pollution level in the water column. One common hemipteran species, *Sigara* sp. exhibited different responses to the environmental variables between different methods.

## 4 DISCUSSION

Existing freshwater bioassessment programs often use higher‐level taxonomic groups, for example, genus or family, due to the practical reasons (e.g., Carlson, Donadi, & Sandin, 2018; Pandey et al., 2017). Consequently, there is still a great deal of uncertainty in determining species‐specific responses of pond ecosystem to various environmental factors. This study reveals the combined effects of pollution levels in sediments and water column as well as pond size on macroinvertebrates identified using both DNA metabarcoding and morphology to provide more precise information for bioassessment and management.

DNA metabarcoding worked especially well for Diptera, because it identified considerably more Diptera species than morphological identification in all ponds. Morphological identification of Diptera larvae, especially Chironomidae, is often very difficult and complicated even for specialists, due to the lack of diagnostic characters (Baloğlu et al., 2018; Dobson, 2013; Silva & Wiedenbrug, 2014). In addition, improper specimen handling leading to the damage of important morphological features, such as scales and bristles, makes morphological identification a difficult task (Chan et al., 2014). The results of the current study are in agreement with the findings of, for example, Ekrem, Stur, and Hebert (2010), Silva and Wiedenbrug (2014) and Lin, Stur, and Ekrem (2018), who found that DNA barcoding is a promising tool for identification of Diptera and Chironomidae. DNA barcoding is also effective in detecting previously unknown diversity (Lin, Stur, & Ekrem, 2015), as shown in our study.

However, for some groups, for example, Oligochaeta and Odonata, DNA metabarcoding identified less species in some samples compared with the morphological identification. The accuracy of DNA metabarcoding identification depends on the “barcode gap,” the existence of a gap between interspecific and intraspecific genetic divergence. Thus, for species complexes where maximum intraspecific distance exceeds the distance to the nearest neighbor, species‐level identification becomes ambiguous (Jinbo, Kato, & Ito, 2011; Sun et al., 2016). There are various reasons resulting in the lack of large interspecific divergence, such as hybridization between closely related species, short time since speciation, and incomplete lineage sorting (Lin et al., 2015). High intraspecific divergence can also affect identification success if they represent cryptic species (i.e., multiple genetic clusters in the reference library have the same name because they cannot be separated morphologically) or are due to infection with endoparasites (e.g., *Wolbachia*) that affect sexual compatibility and fertility resulting in divergent mitochondrial lineages within a species (e.g., Smith et al., 2012). Furthermore, if reference sequences are lacking in established reference databases, DNA metabarcoding identification will also fail (Chan et al., 2014). The missing Oligochaeta, Odonata, and Gastropoda species in our DNA metabarcoding results can be attributed to several reasons. First of all, it can be that our primers did not match well with the species; but this is not plausible for all taxa, as several of the missing species in some samples were detected by DNA metabarcoding in other samples. Secondly, the oligochaete reads could have been missed due to our read abundance threshold of five reads per the sample in order to be included in the analyses; this could mean that oligochaetes were present in the samples, but in very low abundance. Thirdly, the species that were identified morphologically may have not been included in the reference library, and therefore excluded from the DNA metabarcoding results, as we have taken into account only the reads that had a higher than 97% match with the reference library. Last but not least, the samples may not have been preserved well, as Oligochaeta normally need more specialized preservation to keep their DNA available (Rodriguez & Reynoldson, 2011). Thus, DNA metabarcoding gives more species‐level information in general, but results are only as good as the reference library (Hajibabaei, Baird, Fahner, Beiko, & Golding, 2016).

Regarding the (dis)similarity of macroinvertebrate community composition, the taxa analyzed by morphological identification using presence/absence spread all over the space, while the species identified using DNA metabarcoding were clearly divided into several clusters. Results from morphological identification using abundance provided the highest explained variation of macroinvertebrates. However, DNA metabarcoding detected a much higher number of taxa. Therefore, the dimensionality of the DNA metabarcoding data is considerably more complex compared to the morphological data, and an increase in the number of species is normally followed by a decrease in the explained variation.

The correlation matrix showed that PCoA scores extracted from the first axis of DNA metabarcoding were strongly correlated with PCoA scores extracted from the first three axes of morphological identification based on presence/absence, indicating community composition patterns were similar between the DNA metabarcoding and morphological identification based on presence/absence. Despite a correspondence in community composition patterns between datasets from DNA metabarcoding and morphological identification based on presence/absence, there was a lack of significant correlation between the DNA metabarcoding and morphological identification based on abundance, and this could be attributed to the difference in data formats, in which DNA metabarcoding was semi‐quantitative dataset and morphological identification based on abundance was quantitative dataset.

Regarding the results of db‐RDA, it should be mentioned that the water pollution level in our study just reflected a snapshot of the water quality and that concentrations in stormwater ponds vary a lot due to the episodic nature of road runoff. Therefore, the sediment pollution is more reliable as an indicator of pollutant levels in the ponds, and probably much more reliable when comparing the pollutant levels between different ponds. Casey et al. (2007) also demonstrated that the loadings of sediments with pollutants are relatively stable over time.

Our results for the morphological methods are in line with those of Tchakonté et al. (2015), Arimoro, Odume, Uhunoma, and Edegbene (2015), and Hilsenhoff (1988), who found that most dipteran taxa are known as pollution tolerant, normally colonizing polluted waters. However, Diptera in these studies were identified to genus, subfamily, or family level only. Raunio, Paavola, and Muotka (2007) suggested that when chironomid genera are identified to the species level, they should be classified as pollution intolerant, because congeneric species may be different in their tolerance, this being particularly relevant for the species‐rich genera. Different from the results for morphological identification, the results for DNA metabarcoding indicates that species in the highly species‐rich group Diptera show a broader variation in their tolerance to pollution than just classifying them as pollutant tolerant taxa.

The common species from Odonata group, *Libellula quadrimaculata*, that exhibited the same response to environmental variables in DNA metabarcoding and morphological identification belongs to the family Libellulidae, which contains many tolerant species (Villalobos‐Jiménez, Dunn, & Hassall, 2016). Dragonflies and damselflies are normally considered sensitive to different stressors, such as pollutants (Ferreras‐Romero, Márquez‐Rodríguez, & Ruiz‐García, 2009); however, although some sensitive taxa belong to Odonata, the sensitivity/tolerance to a stressor depends on the particular species rather than a larger taxon (Villalobos‐Jiménez et al., 2016). For example, *Aeshna juncea* in our results exhibited positive correlation with pollution level in the sediments and pond size, while negative correlation with the pollution level in the water column. Girgin, Kazanci, and Dügel (2010) have proven that *Aeshna juncea* is highly tolerant of manganese and nickel.

DNA metabarcoding is proposed as a tool to reduce the time and cost necessary for morphological identification: the cost of DNA metabarcoding using high throughput sequencing techniques (HTS) is comparable to or even lower than traditional morphology‐based methods (Hulley et al., 2018; Yu et al., 2012). The use of DNA metabarcoding could also result in substantial reduction in time spent on identification. It normally takes much longer time to process samples and produce data for morphology‐based methods, while the corresponding time for DNA metabarcoding based on HTS is several days due to the higher throughput and no need for sorting individual samples (Tnah et al., 2019).

DNA metabarcoding is a powerful tool for identification of invertebrates (Beermann et al., 2018; Chan et al., 2014; Elbrecht et al., 2017). In our study, comparisons of morphological identification and DNA metabarcoding clearly illustrate that DNA metabarcoding was particularly beneficial for the identification of dipteran species. Since different species within higher taxonomic groups may respond differently to the pollution levels and other environmental variables, such differences are likely to be underestimated or go unnoticed if the taxonomic resolution is low. The results of this study have shown that reliance on morphological methods has limited our perception of the aquatic biodiversity in response to anthropogenic stressors. Even though most families of Diptera have been considered moderately to highly tolerant, most dipteran species vary with respect to pollution tolerance. Application of DNA metabarcoding would greatly increase the number of species identified at a sampling site, thereby providing a more accurate estimate of how aquatic biodiversity responds to anthropogenic stressors. Although the morphological dataset that included abundance provided the best‐explained variation of macroinvertebrates and of environmental variables on the biological community composition, we cannot say that morphological identification including abundance performs significantly better than DNA metabarcoding. This is because DNA metabarcoding gave a significant increase in the number of species, substantially increasing the complexity and dimensionality of the data. Therefore, it is imperative that future research and monitoring apply DNA metabarcoding to ensure that bioassessments do not underestimate the differences in species‐specific responses to environmental factors.

## CONFLICT OF INTEREST

None declared.

## AUTHORS’ CONTRIBUTIONS

All authors contributed to the design of the study. M.M. conducted the molecular laboratory work; M.M. and Z.S. performed data analysis; Z.S. drafted the article, and M.M., E.S., S.R., S.M., and T.E. contributed revisions. All authors gave final approval for publication.

## Data Availability

Whole dataset generated and analyzed during the current study is available in the ENA SRA repository with study name PRJEB30841.
