# Supplementary material for: DNA metabarcoding adds valuable information for management of biodiversity in roadside stormwater ponds
Source: Ecol Evol. 2019 Aug 2;9(17):9712–22. doi: 10.1002/ece3.5503 (PMC6745668; doi:10.1002/ece3.5503)
Supplement: Supplementary file 1 [file ECE3-9-9712-s001.docx]

Supporting Information

DNA metabarcoding adds valuable information for management of biodiversity in roadside stormwater ponds

Zhenhua Sun^a,^*, Markus Majaneva^b^, Ekaterina Sokolova^a^, Sebastien Rauch^a^, Sondre Meland^c,d^, Torbjørn Ekrem^b^

^a^ Chalmers University of Technology, Architecture and Civil Engineering, Water Environment Technology, 412 96 Gothenburg, Sweden

*Corresponding author: [zhenhua.sun@chalmers.se](mailto:zhenhua.sun@chalmers.se), +46317721984

^b^ Norwegian University of Science and Technology, NTNU University Museum, Department of Natural History, 7491, Trondheim, Norway

^c^ Norwegian University of Life Sciences, Faculty of Environmental Sciences and Natural Resource Management, PO 5003, 1432 Ås, Norway

^d^ Norwegian Institute for Water Research (NIVA), Gaustadalléen 21, 0349 Oslo, Norway


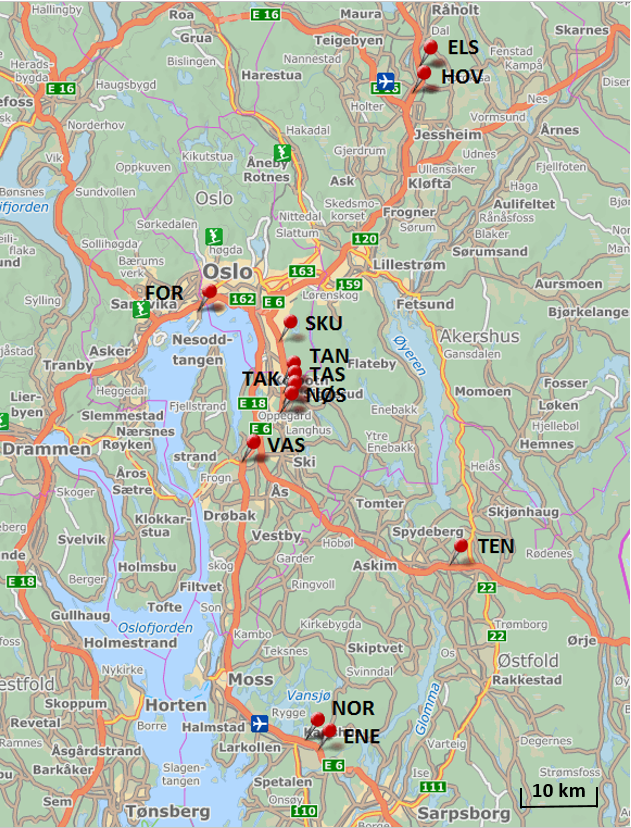


Figure S1. Location of all the studied stormwater ponds (red dots) in the counties of Oslo, Akershus and Østfold. The ponds are: ELS – Elstadmoen, HOV – Hovinmoen, FOR – Fornebu, SKU – Skullerud, TAN – Taraldrud north, TAK – Taraldrud crossing, TAS – Taraldrud south, NØS – Nøstvedt, VAS – Vassum, TEN – Tenor, NOR – Nordby, and ENE – Enebekk.

# Principal Component Analysis (PCA) for pollution levels in sediments and water column

Figure S2. Principal Component Analysis (PCA) for pollution levels in sediments and water column. “S” and “W” represent pollution levels in the sediments and water column, respectively. The first axis explained 48% and the second axis explained 24% of the variation. . The following abbreviations are used for 12 ponds: ELS – Elstadmoen, HOV – Hovinmoen, FOR – Fornebu, SKU – Skullerud, TAN – Taraldrud north, TAK – Taraldrud crossing, TAS – Taraldrud south, NØS – Nøstvedt, VAS – Vassum, TEN – Tenor, NOR – Nordby, and ENE – Enebekk.

# Species in common

In total, 56 species were identified using both DNA metabarcoding and morphology (*Eiseniella tetraedra, Lumbriculus variegatus, Slavina appendiculata, Stylodrilus heringianus, Limnodrilus hoffmeisteri, Ophidonais serpentine, Tubifex tubifex, Erpobdella octoculata, Helobdella stagnalis, Theromyzon tessulatum, Planorbis planorbis, Armiger crista, Radix balthica, Coenagrion hastulatum, Enallagma cyathigerum, Coenagrion pulchellum, Pyrrhosoma nymphula, Lestes sponsa, Erythromma najas, Aeshna grandis, Aeshna juncea, Libellula quadrimaculata, Leucorrhinia dubia, Centroptilum luteolum, Cloeon simile, Caenis horaria, Leptophlebia vespertina, Leptophlebia marginata, Ephemera vulgata, Ephemera danica, Nemoura cinerea, Callicorixa praeusta, Corixa dentipes, Notonecta glauca, Notonecta maculate, Notonecta lutea, Hesperocorixa linnaei, Hesperocorixa sahlbergi, Gerris odontogaster, Hydrometra gracilenta, Agrypnia varia, Agraylea sexmaculata, Holocentropus dubius, Holocentropus picicornis, Limnephilus rhombicus, Limnephilus borealis, Limnephilus extricates, Limnephilus fuscicornis, Nemotaulius punctatolineatus, Plectrocnemia conspersa, Phryganea bipunctata, Elophila nymphaeata, Sialis fuliginosa, Chaoborus crystallinus, Chaoborus flavicans,* and *Chaoborus obscuripes*). Richness differences were greatest for Diptera (168 additional species by identification with DNA metabarcoding, 3 species in common), followed by Coleoptera (23 additional species by DNA metabarcoding, no species in common; all taxa identified by morphology were to genus or family level), Hemiptera (21 additional species by DNA metabarcoding, 9 species in common), Trichoptera (7 additional species by DNA metabarcoding, 11 species in common), Lepidoptera (7 additional species by DNA metabarcoding, 1 species in common) and Oligochaeta (4 additional species by DNA metabarcoding, 7 species in common).

# Redundancy analysis (RDA) for the Diptera group

The results of db-RDA for the DNA metabarcoding dataset showed that pollution levels in the water column and sediments, as well as pond size explained 32% of variances (first axis: F = 1.4, p = 0.032; all axes: F = 1.3, p = 0.036) in the macroinvertebrate community composition, and the first two axes explained 15% and 9%, respectively (Figure S3A). For the morphological dataset based on presence/absence, the three variables explained 25% of variances (first axis: F = 1.2, p = 0.565; all axes: F = 0.9, p = 0.656); for the morphological dataset based on abundance, these three environmental variables explained 30.8% of variances (first axis: F = 1.5, p = 0.443; all axes: F = 1.2, p = 0.288), and the first two axes explained 16% and 13%, respectively. However, all axes based on datasets derived from morphological identifications were not statistically significantly correlated with the dipteran community composition (Figure S3B and S3C).

B)

A)

C)

Figure S3. The relationships between dipteran community composition and pollution levels in the water column and sediments, as well as pond size. A) Results obtained from db-RDA for the DNA metabarcoding dataset. B) Results obtained from db-RDA for the morphological dataset based on presence/absence. C) Results obtained from db-RDA for the morphological dataset based on abundance. The purple arrows represent different species; the red arrows represent environmental variables, in which WaterPol and Sediment represent pollution levels in the water column and sediments, respectively.

Table S1. Size of the studied stormwater ponds

| Ponds | Pond surface area (m^2^) |
| --- | --- |
| Skullerud (SKU) | 910 |
| Taraldrud north (TAN) | 780 |
| Taraldrud crossing (TAK) | 1400 |
| Taraldrud south (TAS) | 474 |
| Nøstvedt (NØS) | 340 |
| Vassum (VAS) | 363 |
| Nordby (NOR) | 89 |
| Enebekk (ENE) | 132 |
| Tenor (TEN) | 480 |
| Fornebu (FOR) | 480 |
| Hovinmoen (HOV) | 422 |
| Elstadmoen (ELS) | 741 |

# Distance used in principal coordinates analysis and distance-based redundancy analysis

The Sørensen coefficient (Sørensen, 1948) is defined as

$$S_{1,2}=\frac{2a}{2a+b+c}$$

where *a* is the number of species present in both cases being compared, *b* is the number of species present in case 1 only, and *c* is the number of species present in case 2 only. The similarity of identical cases equals 1 and the similarity of cases without same species equals 0 (Šmilauer and Lepš, 2014).

The Bray-Curtis distance (Bray and Curtis, 1957) is defined as

$${PS}_{1,2}=\frac{2\sum_{k=1}^{m} min(Y_{1k},Y_{2k})}{\sum_{k=1}^{m} (Y_{1k}+Y_{2k})}$$

$$PD=1-PS (or PD=100-PS, when multiplied by 100)$$

where PS is percentage similarity; PD is percentage dissimilarity. For a data matrix with *m* species, with the value of *k*-th species in the *i*-th case written as *Y_ik_*.

# DNA metabarcoding

The first PCR reactions were assembled in 25μL volumes containing 2 μL of 1:10 diluted DNA template, 17.3 μL molecular biology grade water, 2.5 μL 10× reaction buffer (200 mM Tris HCl, 500 mM KCl, pH 8.4), 1 μL MgCl_2_ (50 mM), 1 μL dNTPs mix (10 mM), 0.5 μL forward primer (10 mM), 0.5 μL reverse primer (10 mM), and 0.2 μL Invitrogen’s Platinum Taq polymerase (5 U/μL). The PCR conditions were initiated with heated lid at 94°C for 5 min, followed by a total of 35 cycles of 94°C for 40 s, 46°C for 1 min, and 72°C for 20 s, and a final extension at 72°C for 2 min, and hold at 10°C. PCR products were visualized on a 1.5% agarose gel and purified, using Qiagen’s MiniElute PCR purification kit according to manufacturer’s instructions. The two fragments as well as PCR and DNA extraction negative triplicates were pooled to represent each DNA sample. Samples were dual indexed in a second PCR reaction with the same reagent concentrations and PCR conditions as the first PCR reaction except, using Nextera XT Index 1 and 2 primers (FC-131-1002, Illumina, Inc., San Diego, CA, USA), in a total volume of 50 µL and only 10 cycles. The indexed samples were purified, using MinElute PCR purification kit and pooled in two sequencing libraries. The libraries were sequenced, using the 600-cycle V3 Illumina MiSeq sequencing kit (MS-102-3003).

The resulting raw reads were processed initially, using mothur v.1.39.5 (Kozich et al., 2013). The two amplified fragments were divided into separate files based on presence of primers (no mismatch in primer sequence) using fastq.info command. In the next step, make.contigs command was used to combine forward and reverse reads with minimum overlap of 25 bases. All merged F230 reads that were shorter than 250 bases and longer than 310 bases and all merged BFR2 reads shorter than 430 and longer than 490 bases, as well as merged reads with ambiguous bases were removed, using the command screen.seqs. The trim.seqs command trimmed off the primers at the beginning and in the end of the reads. After the quality control, the resulting good quality reads were processed further, using usearch v.10.0.240_win32 (Edgar, 2010). The exact duplicates were removed, using the command -fastx_uniques, and the reads were clustered to 97-% OTUs, using -cluster_otus command. Chimeras were identified and removed in this step. The above steps were done for each sample and the resulting OTU-fasta files were pooled to F230 and BFR2 files, using merge.files in mothur. Then, the resulting pooled OTU files were de-replicated, using - fastx_uniques command in usearch, and the final OTUs (two fragment separately) were clustered, using -cluster_otus. The abundance of each OTU in each sample was searched against a pooled good quality read file (made by merging the uniques.fasta files after the first -fastx_uniques command in usearch), and an OTU table was compiled, using -otutab command in usearch.

The OTUs were identified taxonomically in two steps. First, they were searched against the NCBI non-redundant nucleotide database, using the BLAST 2.6.0+ (Zhang et al., 2000) September 15 2017 (F230 fragment) and September 19 2017 (BFR2 fragment). Taxonomic assignment of an OTU was done, using the lowest common ancestor algorithm in MEGAN 6.8.19 (minimum bit score 100, top percentage 8.0 and minimum support 1; Huson et al. (2016)). A low minimum bit score was used to identify all possible Metazoa OTUs that were further identified in the BOLD System v4 Species Level Barcode Records September 15 (F230) and 19 (BFR2) 2017. The OTUs that matched with an invertebrate species in the BOLD v.4 Species Level Barcode Records were kept, and all OTUs matching with the same species name were merged leaving 599 invertebrate species level OTUs (called DNA-species hereafter).

# References

EDGAR, R. C. 2010. Search and clustering orders of magnitude faster than BLAST. *Bioinformatics,* 26.

HUSON, D. H., BEIER, S., FLADE, I., GÓRSKA, A., EL-HADIDI, M., MITRA, S., RUSCHEWEYH, H.-J. & TAPPU, R. 2016. MEGAN Community Edition - Interactive Exploration and Analysis of Large-Scale Microbiome Sequencing Data. *PLOS Computational Biology,* 12**,** e1004957.

KOZICH, J. J., WESTCOTT, S. L., BAXTER, N. T., HIGHLANDER, S. K. & SCHLOSS, P. D. 2013. Development of a Dual-Index Sequencing Strategy and Curation Pipeline for Analyzing Amplicon Sequence Data on the MiSeq Illumina Sequencing Platform. *Applied and Environmental Microbiology,* 79**,** 5112-5120.

ZHANG, Z., SCHWARTZ, S., WAGNER, L. & MILLER, W. 2000. *Greedy Algorithm for Aligning DNA Sequences*.
